# Prognostic Outcomes of Tall Cell Variant Papillary Thyroid Cancer: A Meta-Analysis

**DOI:** 10.4061/2010/325602

**Published:** 2010-07-26

**Authors:** Scharukh Jalisi, Tiffiny Ainsworth, Michael LaValley

**Affiliations:** ^1^Department of Otolaryngology, Head and Neck Surgery, Boston University School of Medicine, 820 Harrison Avenue, FGH4, Boston MA 02118, USA; ^2^Department of Otolaryngology, University of Connecticut, Farmington, CT 06262, USA; ^3^Department of Biostatistics, Boston University School of Public Health, Boston, MA 02118, USA

## Abstract

*Objective*. To evaluate the prognosis of tall cell variant (TCV) compared to usual variant (UV) papillary thyroid cancer by comparing disease-related mortality and recurrence data from published studies. *Methods*. Ovid MEDLINE keyword search using “tall cell variant papillary thyroid cancer” was used to identify studies published in English that calculated disease-related mortality and recurrence rates for both TCV and UV. *Results*. A total of 131 cases of tall cell variant papillary thyroid cancer were reviewed. The combined odds ratio of recurrence for TCV compared to UV is 4.50 with a 95% confidence interval from 2.90 to 6.99. For disease-related mortality, the combined odds ratio for TCV was compared to UV of 14.28 with a 95% confidence interval from 8.01 to 25.46. *Conclusion*. Currently published data suggests that TCV is a negative prognostic indicator in papillary thyroid cancer and requires aggressive therapy. This meta-analysis provides the largest prognostic data series on TCV in the literature and clearly identifies the need for accurate pathological identification of TCV and its further study as an independent prognostic factor.

## 1. Introduction

Tall cell variant papillary thyroid carcinoma (TCV) was first described in 1976 by Hawk and Hazard as an aggressive histological variant of papillary thyroid cancer [1]. TCV is defined as tall papillary epithelial cells with a height at least twice the width and basement membrane oriented nuclei comprising at least 30% of the cells within the tumor [1–3]. Usual variant papillary thyroid cancer describes conventional papillary thyroid carcinoma composed of papillary epithelial cells arranged in papillae with fibrovascular cores without excessive height compared to cell width. The published incidence of TCV is between 3.8% and 10.4% [1, 4–10]. Unlike the usually good prognosis of usual variant papillary thyroid cancer (UV), TCV histology is generally reported as an independently poor prognostic factor [1, 3, 5, 6, 8, 9]. 

More recently reported case series do not show that TCV histology adversely affects prognosis [7, 10, 11]. One such large retrospective study concluded the poor prognosis of TCV tumors was not particularly related to histology but to factors such as stage and grade [10]. Another case series found the higher rate of recurrence in TCV was strictly associated with age over 50 [7]. Although these more recent studies improved on previously reported studies by increasing the sample sizes, the number of TCV cases still remain small in comparison to the UV group. A reason for the low TCV numbers could be misclassification bias where the diagnosis of TCV is missed by routine pathologic examination. As first described in a case series of 162 papillary thyroid cancers, initial routine pathological examination failed to detect 8 out of 11 cases of TCV [4]. The additional eight cases were discovered only by an experienced pathologist with a special interest in thyroid pathology and familiar with TCV histology. As larger sample sizes are studied, the potential for misclassification bias also increases. This may lead to erroneous comparisons of recurrence and mortality data between TCV and UV. 

The purpose of this meta-analysis is to evaluate the prognostic impact of TCV histology in papillary thyroid cancer in currently published studies by comparing TCV and UV disease-related mortality and recurrence data. We hypothesize that the cumulative published evidence supports TCV as a negative prognostic factor as measured by a greater disease-related mortality and recurrence rate than UV.

## 2. Methods

A systematic literature review was conducted on May 23, 2007 utilizing the Ovid search engine to access the MEDLINE database. Articles from January 1, 1980 to May 23, 2007 were evaluated. The term “tall cell variant papillary thyroid cancer” was used as a keyword search and yielded 108 articles. The titles and abstracts were carefully reviewed and those evaluating the prognostic implication of TCV histology in papillary thyroid cancer were included for review. The papers were evaluated based on predetermined inclusion and exclusion criteria and their references were reviewed for additionally relevant papers. The primary inclusion criterion was studies that compared prognostic factors in TCV to UV papillary thyroid cancer. Exclusion criteria included those with total sample size less than or equal to 15, non-English reports, and the absence of recurrence and disease-related mortality data for both TCV and UV groups. The MEDLINE and bibliography search after application of the inclusion and exclusion criteria yielded six reports that were included in our study (Table 1).

All statistical analyses were performed by a single statistician. Both odds ratios and 95% confidence intervals for both recurrence and disease-related mortality were calculated for each of the studies. In the case of a 0 count in a study, 0.5 was added to all the counts for that study to allow calculation of an odds ratio and 95% confidence interval. The next step determined the combined odds ratio of all six studies for recurrence and disease-related mortality. Both the fixed effects Cochran-Mantel-Haenszel and a random effects method for binary data were used to calculate combined odds ratios. A fixed effects method gives each study a weighting in accordance to the precision of its estimate whereas a random effects method accounts for variation between studies by giving a relatively larger weight to the less precise studies. 

For both the odds of recurrence and mortality, a test for heterogeneity that accompanies the fixed effects, Cochran-Mantel-Haenszel model was significant (*P* =  .023 and *P* =  .033, resp.) suggesting that between-study variability was too great for a fixed effects model. As a result, the random effects method to determine the odds ratio was preferred to account for the extra variation between studies.

## 3. Results

After application of our inclusion and exclusion criteria, six articles were available for review (Table 1). The study by Segal et al. and Terry et al. was included due to the presence of recurrence and mortality data. The total number of TCV cases for review in our study was 131. This allows for our study to have the largest number of TCV cases available for evaluation in the literature to the best of our knowledge. The mean age at diagnosis of TCV was greater than 45 years. No data on gender predilection could be determined from the studies. Mean tumor size was comparable to the tumor size in UV papillary thyroid cancer. Reviewing the data from [3, 8–10], the TCV patients had a higher rate of extrathyroidal extension (cumulative average of 60.33% patients) and higher rate of distant metastases at diagnosis (cumulative average of 15%). In addition, the cumulative average of lymph node metastases in TCV cases is 58.12% versus 34.5% in UV cases. The percentage of recurrence and disease-related mortality is also noted to be higher in TCV group versus UV group. The cumulative average recurrence in TCV group is 42.5% versus 9.8% in UV group. The cumulative average disease-related mortality in TCV group is 23.6% versus 1.5% for UV group. 

The odds ratios calculated by this study for recurrence and disease-related mortality are provided in Table 2. All of the individual study's odds ratios demonstrate a greater odds of recurrence in the TCV subjects compared to the UV subjects. The odds ratios are statistically significant in all the studies except the Prendiville et al. study [8]. All the calculated odds ratios for individual study disease-related mortality similarly demonstrated an increased rate of disease-related mortality in TCV subjects compared to UV subjects; however, only three of the six studies [6, 9, 10] had statistically significant odds ratios where the confidence interval did not include one. 

The combined odds ratios for recurrence and disease-related mortality using both the fixed and random effects methods are provided in Table 3. The test for heterogeneity (*P* =  .023) that accompanies the random effects method in determining the odds of recurrence indicates the random effect method would be preferable to account for the extra between-study variation by giving a relatively larger weight to the less precise studies. Although both combined odds ratios of recurrence demonstrate a greater rate of recurrence in the TCV subjects compared to UV subjects, the preferred random effects model odds ratio suggests that recurrence occurs with 4.50 times greater odds in TCV tumors versus UV tumors and that this is statistically significant (95% CI 2.90–6.99).

Similarly, the combined odds of disease-related mortality is greater for TCV subjects compared to UV subjects regardless of whether a fixed versus random effects method is used. Using the preferred random effects model, the odds of disease-related mortality in TCV patients are 14.28 times greater than UV patients. This is also statistically significant (95% CI: 8.01–25.46). 

## 4. Discussion

TCV is a negative prognostic indicator in papillary thyroid cancer. By performing a meta-analysis on published research, we hoped to overcome the limitations of small sample sizes of individual articles to examine the impact of such a rare histological subtype on prognosis. The total number of TCV cases in our analysis are 131. To summarize, we found TCV recurs with 4.50 times greater odds than UV. This is statistically significant with a 95% confidence interval between 2.90 and 6.99. Additionally, we found TCV has a 14.28 times greater disease-related odds of mortality compared to UV. This is statistically significant with a 95% confidence interval between 8.01 and 25.46. We also noted higher trends in lymph node metastasis, distant metastasis, and extrathyroidal extension in TCV patients versus UV patients. 

The adverse effects of TCV on prognosis, as represented by our study, necessitate a careful and perhaps more aggressive approach than typically followed with UV. Whereas partial thyroidectomy (defined as thyroid lobectomy with or without isthmusectomy) is an acceptable conservative approach to patients with low-risk UV for recurrence and mortality (by AMES criteria), our research suggests a more aggressive approach for TCV (e.g., total thyroidectomy with central neck dissection) may be indicated. The problem at hand is that TCV is detected at pathological evaluation, which occurs *after* the initial thyroid surgery has been performed. Hence, we propose that if a newly diagnosed patient with TCV has undergone partial thyroidectomy, then the patient should return to the operating room for at least a completion thyroidectomy and central neck dissection followed by radioactive iodine ablation of residual tissue. If a total thyroidectomy was performed at initial surgery, then careful consideration should be given for return to the operating room for a central neck dissection (depending on surgeon experience) followed by radioactive iodine ablation versus radioactive iodine ablation alone. If the tumor is not radioactive iodine avid, then early intervention with external beam radiation should be considered. Additional issues, such as longer follow-ups and extensive screening for distant metastasis, may also be warranted. A recent review of papillary thyroid cancer variants recommended an aggressive approach to TCV [13] with at least a total thyroidectomy.

In a recent matched-pair analysis comparing TCV patients to the matched UV cohort, 5-year disease specific survival was poorer in the TCV cohort (81.9% versus 91.3%, *P* =  .049). The number of deaths in the TCV cohort was higher than in the matched Papillary thyroid cancer cohort (*P* =  .043) [14]. This study further affirms the results of our study showing higher rate of recurrence and mortality in TCV patients.

Our results stress the necessity of accurate pathological diagnosis. Since the discovery of TCV in 1976, the ability to study and evaluate the prognostic implications of histology in papillary thyroid cancer resides solely on accurate pathological identification. Hence, there is no substitute for an experienced pathologist in the management of this disease.

There are several limitations in our study. Firstly, we have large confidence intervals which reflect the variation between studies and the relatively small number of studies included in the meta-analysis. This is also reflected in the test of heterogeneity that we performed that directed us to use the random effects model rather than the fixed effects model. In addition, the articles by Segal et al. [6] and Terry et al. [7] did not have data on patient age, tumor size, lymph node metastasis, extrathyroidal extension, or distant metastases. These articles were included due to the presence of recurrence and disease-related mortality data. Good faith efforts were made to contact the authors for this data without success. 

## 5. Conclusions

In conclusion, the data supports TCV as a poor prognostic factor as determined by disease-related mortality and recurrence rates. As a result, this data supports early aggressive surgical and adjuvant radioactive iodine management and the necessity for accurate pathologic diagnosis. It unequivocally supports the absolute need for further study of TVC as an independent prognostic factor in future studies on papillary thyroid cancer.

## Figures and Tables

**Table 1:** 

Study	Mean age	Mean tumor size (cm)	% positive lymph nodes	% extra-thyroidal extension	% distant metastasis	% recurrence	% disease-related mortality
*Johnson*							
TCV (*n* = 12)	49.4	2.8	75	42	17	58.3	25
NV (*n* = 12)	48.3	2.3	42	0	0	8.3	0

*Michels*							
TCV (*n* = 56)	50	2	37.5	39	8	14.3	12.5
NV (*n* = 503)	45.6	1.5	23	9	2.6	5.4	1.6

*Moreno Egea*							
TCV (*n* = 5)	65.4	≥2.86*	80	100	20	80	20
NV (*n* = 85)	41.5	NA**	34.1	14.1	NA**	16.5	1.2

*Prendiville*							
TCV (*n* = 20)	49.6	2.1	40	65	NA**	20	5
NV (*n* = 1355***)	35.7	2.5	39	8	NA**	15	2

*Segal*							
TCV (*n* = 19)	NA**	NA**	NA**	NA**	NA**	47.4	73.7
NV (*n* = 223)	NA**	NA**	NA**	NA**	NA**	9.9	3.6

*Terry*							
TCV (*n* = 19)	NA**	4.2	NA**	NA**	NA**	35.3	5.9
NV (*n* = 118)	NA**	2.8	NA**	NA**	NA**	3.8	0.8

*The size of two tumors reported >4 cm.

**Not Available.

***Article used NV data from Mazzaferri and Jhiang [12].

**Table 2:** 

Study	Recurrence Odds Ratio [95% Confidence Interval]	Disease-related Mortality Odds Ratio [95% Confidence Interval]
Johnson	15.4 [1.47–160.97]	9.21 [0.42–200.34]
Michels	2.94 [1.26–6.83]	8.84 [3.07–25.41]
Moreno Egea	20.29 [2.11–195.38]	21.00 [1.10–400.30]
Prendiville	1.42 [0.47–4.29]	2.59 [0.33–20.4]
Segal	8.22 [3.02–22.40]	75.25 [21.75–260.36]
Terry	13.85 [3.64–52.76]	8.19 [0.49–137.36]

**Table 3:** 

	Recurrence [95% confidence interval]	Disease-related mortality [95% confidence interval]
Fixed effects model	4.61* [2.94–7.22]	16.47^#^ [8.86–30.60]
Random effects model	4.50 [2.90–6.99]	14.28 [8.01–25.46]

*Test for between-study heterogeneity statistically significant (*P* =  .023).

^#^Test for between-study heterogeneity statistically significant (*P* =  .033).
